# Caractéristiques de l'association diabète type 2 et hypertension artérielle chez le sujet âgé de 65 ans et plus

**DOI:** 10.11604/pamj.2013.14.100.1880

**Published:** 2013-03-12

**Authors:** Khadija Diyane, Nawal El Ansari, Ghizlane El Mghari, Karim Anzid, Mohamed Cherkaoui

**Affiliations:** 1 Service d'endocrinologie, diabétologie et maladies métaboliques, Laboratoire de recherche de pneumo-cardio-immunopathologie et métabolisme PCIM, Faculté de Médecine et de Pharmacie de Marrakech, Université cadi Ayyad. CHU Mohamed VI. Rue el mostachfa, Gueliz, 40 000, Marrakech, Maroc; 2 Laboratoire d’Écologie Humaine. Faculté des sciences semlalia. Université Cadi Ayyad. Marrakech, Maroc

**Keywords:** Complications dégénératives, diabète type 2, dyslipidémie, hypertension artérielle, sujet âgé, degenerative complications, Type 2 diabetes, dyslipidemia, hypertension, elderly

## Abstract

**Introduction:**

L'HTA du diabétique âgé est particulière par sa fréquence et sa gravité. Cette association HTA-diabète type 2 (DT2) est particulièrement fréquente chez la personne âgée, et responsable d'une majoration du risque cardiovasculaire et d'une accélération de l'atteinte dégénérative du diabète.

**Méthodes:**

Etude descriptive, concernant 100 patients diabétiques de type 2 hypertendus âgés de 65 ans ou plus, suivis au service d'endocrinologie-diabétologie du CHU de Marrakech, du mois de Novembre 2010 au mois de Juillet 2011. Le logiciel SPSS version 18 a été utilisé pour l'analyse statistique.

**Résultats:**

Le sex-ratio des patients étudiés était de 0,26, l’âge moyen était de 69,2 ±; 4,3 ans, l'ancienneté du diabète était de 9,3 ±; 6,7 ans. Le diagnostic du diabète précédait celui de l'HTA dans 67,7% des cas. Seulement 4,2% avaient une HbA1c ≤ 6,5%. 60% des patients avaient une HTA de grade I. L'IMC moyen était de 28,1 ±; 4,6 kg/m^2^. La dyslipidémie était présente chez 59,6% de nos patients avec essentiellement une hypoHDLémie (75,9%). La macroangiopathie était observée chez 40% des patients avec essentiellement une cardiopathie ischémique (29%). Elle était significativement plus fréquente chez les patients ayant une HbA1c supérieure à 9%, LDL-c ≥ 1 g/l et une hypoHDLémie. La microangiopathie présente dans 82% des cas était significativement en relation avec l'HbA1c, le DFG et le taux des triglycérides.

**Conclusion:**

Une prise en charge complète du risque cardio-vasculaire chez les sujets âgés se heurte à des problèmes objectifs en pratique courante, en particulier, la polymédication, source d'une mauvaise compliance et donc de mauvais résultats. Mots clés: Complications dégénératives, Diabète type 2, Dyslipidémie, Hypertension artérielle, Sujet âgé.

## Introduction

Le diabète est une pathologie fréquente chez les sujets âgés, sa prévalence atteint 10 à 20% après 65 ans. Le vieillissement de la population est un des facteurs explicatifs de cette véritable épidémie attendue [1]. Par ailleurs, l'hypertension artérielle (HTA) est associée au diabète de type 2 (DT2) dans 80% des cas [2], elle est plus fréquente particulièrement chez les personnes âgées diabétiques. Cette association HTA-diabète est responsable d'une majoration du risque cardiovasculaire [3] avec survenue des évènements morbides cardiovasculaires et accélération de l'altération de la fonction rénale. Le présent travail, se fixe comme objectif d’étudier les caractéristiques épidémiologiques, cliniques, biologiques et thérapeutiques de l'association HTA-DT2.

## Méthodes

Il s'agit d'une étude transversale prospective, concernant des patients diabétiques de type 2, hypertendus, âgés de 65 ans ou plus; recrutés de façon accidentelle au service d'Endocrinologie-Diabétologie de l'hôpital Ibn Tofail du CHU Mohammed VI de Marrakech que ce soit en hospitalier ou en consultation. L’étude s’étendait du mois de novembre 2010 au mois de juillet 2011. Chaque patient a bénéficié d'un interrogatoire, d'un examen physique, d'un bilan biologique et dégénératif. Le logiciel SPSS version 18.0 a été utilisé pour les analyses statistiques. Une valeur de p ≤ 0.05 définissait le seuil de signification statistique.

## Résultats

Cent patients ont été inclus dans l’étude. Leurs caractéristiques générales sont résumées dans les Tableau 1 et Tableau 2. Le sex-ratio était de 0,26. L'HbA1c moyenne était 9,7 ±; 2,1%. La dyslipidémie était présente chez 59,6% des cas. Il s'agissait essentiellement d'une hypoHDLémie (75,9%), et d'une hyper LDL-c (51,7%).

**Tableau 1:** Les caractéristiques générales de la population d’étude

				Complications dégénératives						
				Avec			Sans			
	n	µ	σ	n	Μ	Σ	n	µ	σ	*P*
**Age (ans)**	100	69,2	4,3	87	69,2	4,3	13	68,8	4,4	0,649
**Ancienneté du diabète (ans)**	99	9,3	6,7	86	10,3	6,4	13	2,4	3,3	0,000
**Ancienneté de l'HTA**	99	5,7	4,9	86	5,7	4,7	13	6,2	6,2	0,770
**IMC (kg/m** ^**2**^ **)**	81	28,1	4,6	70	28,3	4,7	11	27,0	4,1	0,431
**Tour de taille TDT (cm)**	48	102,3	8,8	43	102,8	8,9	5	97,8	6,7	0,141
**Cholesterol total (mg/dl)**	59	2,0	0,5	52	1,9	0,6	6	2,2	0,5	0,345
**HDLc (mg/dl)**	58	0,4	0,1	51	0,4	0,1	6	0,5	0,1	0,246
**LDLc (mg/dl)**	58	1,1	0,4	51	1,1	0,5	6	1,1	0,5	0,845
**TG (mg/dl)**	61	1,5	0,6	52	1,4	0,6	7	1,5	0,3	0,432
**HbA1c (%)**	80	9,7	2,1	68	9,9	2,0	9	8,6	2,1	0,092
**DFG (ml/min/1,73m** ^**2**^ **)**	44	84,2	31,0	35	88,1	27,7	6	87,9	34,8	0,699
**Microalbuminurie (mg/l)**	100	18,6	34,3	84	19,3	33,3	13	13,7	41,3	0,207

(n)= ffectif; (µ)=moyenne; (σ)=écart

**Tableau 2:** La proportion des sujets ayant les caractéristiques suivantes

	N	%	95% CI	
			Borne inférieure	Borne supérieure
**Durée du diabète> 5 ans**	99	70%	60,4%	78,1%
**Sexe féminin**	100	79%	70,0%	85,8%
**Macroangiopathies:**	100	40%	30,9%	49,8%
**Cardiopathie ischémique**	100	29%	21,0%	38,5%
**AVC et ou AIT**	100	11%	6,3%	18,6%
**AOMI**	100	8%	4,1%	15,0%
**Microangiopathies:**	100	82%	73,3%	88,3%
Neuropathie périphérique	100	65%	55,3%	73,6%
Neuropathie autonome	100	38%	29,1%	47,8%
Rétinopathie diabétique	100	31%	22,8%	40,6%
Néphropathie diabétique	100	28%	20,1%	37,5%
**DFG < 60 ml/min/1,73m** ^**2**^	44	23%	13,0%	37,3%
**Grade HTA:**				
I	100	60%	50,2%	69,1%
II	100	33%	24,6%	42,7%
III	100	7%	3,4%	13,7%
**Hypertension artérielle:**	97	97%	91,4%	99,0%
systole-diastolique	100	60%	50,2%	69,1%
Systolique	100	32%	23,7%	41,7%
Diastolique	100	5%	2,2%	11,2%
**surpoids**	97	41,2%	31,9%	51,1%
**Obésité**	97	28,9%	20,8%	38,6%
**Répartition centrale des graisses:**	48	79,9%	66,6%	88,8%
≥80 chez la femme	42	100%	91,6%	100,0%
≥94 chez l'homme	6	83,3%	43,6%	97,0%
**Dyslipidémie:**	99	59,6%	49,8%	68,7%
LDL≥1	58	51,7%	39,1%	64,0%
HDL≤ 0,5mg/dl	58	75,9%	63,5%	85,1%
TG≥ 2mg/dl	61	14,8%	8,0%	25,8%
**Sédentarité**	100	70%	60,4%	78,1%

Les complications dégénératives étaient associées à une ancienneté moyenne du diabète de 10,3 ±; 6,4 ans, versus 2,4 ± 3,3 ans pour le groupe sans complications dégénératives. La différence n’était pas statistiquement significative pour les autres paramètres (Tableau 1).

La macroangiopathie était observée chez 40% des patients avec essentiellement une cardiopathie ischémique (29%) (Tableau 2). Ces atteintes étaient significativement plus fréquentes chez les patients ayant une HbA1c supérieure à 9%, LDL-c ≥ 1 g/l et une hypoHDLémie. L'artériopathie oblitérante des membres inférieurs était plus fréquente chez les patients ayant une hypoHDLémie (Tableau 3). L'effet du contrôle de la tension artérielle n’était pas statistiquement significatif pour la macroangiopathie.

**Tableau 3:** Relation entre la macroangiopathie et les autres facteurs de risque associés

		CMI		AVC		AOMI		
	N	n	%	n	%	n	%	P
**Age (ans)**								
65-69,9	53	24	45,3	9	17,0	20	37,7	0,648
70-74,9	14	6	42,9	3	21,4	5	35,7	
>75	22	14	63,6	3	13,6	5	22,7	
**HbA1c (%)**								
< 6,5	3	0	0,0	3	100,0	0	0,0	0,000
6,5 - 9	21	18	85,7	3	14,3	0	0,0	
> 9	48	14	29,2	9	18,8	25	52,1	
**IMC (kg/m** ^**2**^ **)**								
20-24,9	22	14	63,6	3	13,6	5	22,7	0,238
25-29,9	45	16	35,6	9	20,0	20	44,4	
>30	18	10	55,6	3	16,7	5	27,8	
**Dyslipidémie**								
Oui	62	30	48,4	12	19,4	20	32,3	0,648
Non	27	14	51,9	3	11,1	10	37,0	
**LDL**								
LDL < 1g/l	10	10	100,0	0	0,0	0	0,0	0,048
LDL ≥ 1g/l	38	22	57,9	6	15,8	10	26,3	
**HDL**								
HDL < 0,5g/l	39	26	66,7	3	7,7	10	25,6	0,039
HDL ≥ 0,5g/l	9	6	66,7	3	33,3	0	0,0	
**TG**								
TG < 2g/l	46	30	65,2	6	13,0	10	21,7	0,438
TG ≥ 2g/l	4	4	100,0	0	0,0	0	0,0	
**DFG(ml/min/1,73m2)**								
<60	2	2	100,0	0	0,0	0	0,0	0,129
≥ 60	29	8	27,6	6	20,7	15	51,7	
**Activité physique**								
Inactif/Irrégulière	80	40	50,0	15	18,8	25	31,3	0,232
Régulière	9	4	44,4	0	0,0	5	55,6	

n, effectif;%, pourcentage; CMI, Cardiomyopathie ischémique; AVC, Accident vasculaire cérébral ; AOMI, Artériopathie oblitérante des membres inférieurs.

L'atteinte microangiopathique était présente dans 82% des cas, avec essentiellement une neuropathie périphérique (65%) (Tableau 2). Elle était corrélée à l'HbA1c, au DFG et à l'hypertriglycéridémie.

## Discussion

L'HTA et le DT2 représentent deux grands problèmes de santé publique à l’échelle mondiale, de par leur fréquence, la nécessité d'un suivi et d'un traitement médicamenteux à vie, et de par leurs complications vasculaires. Ces deux pathologies, associées dans 80% des cas, sont plus fréquentes particulièrement chez les personnes âgées, avec un pic situé entre 66-69 ans, contribuant à la majoration du risque cardiovasculaire [2, 3]. D'après les données de l’étude UKPDS, un meilleur contrôle tensionnel permet de réduire de 24% la morbi-mortalité cardiovasculaire, et de 37% les complications microangiopathiques [4]; le contrôle strict de la glycémie est associé quant à lui à une réduction de 12% de la morbi-mortalité globale et de 25% de l'atteinte microangiopathique. Des analyses économiques ont par ailleurs montré qu'un contrôle strict de la PA chez le diabétique avait un meilleur rapport coût/efficacité qu'un contrôle strict de la glycémie [5]. Dans notre étude l'effet du contrôle de la tension artérielle n’était pas statistiquement significatif pour la macroangiopathie.

Un profil lipidique athérogène est habituellement observé chez le patient diabétique; une étude Japonaise a retrouvé une corrélation positive entre la pression artérielle et l'hypoHDLémie chez des patients hypertendus diabétiques âgés de plus de 50 ans [6]. Dans notre étude, l'hypoHDLémie était observée dans 75,9% des cas et le LDL-c était supérieur à 1g/l dans 51,7% des cas [7, 8].

Les complications dégénératives étaient associées à une ancienneté moyenne du diabète de 10,3 ±; 6,4 ans avec une prévalence des complications macroangiopathiques dans 40% des cas; ce qui rejoint les données de la littérature. Dans cette étude les facteurs corrélés à la présence de complications macroangiopathiques chez les sujets âgés hypertendus sont le taux élevé d'HbA1c, et de LDL-c et le taux bas de HDL-c. Dans la série de Fradi l'incidence de la macroangiopathie était corrélée à l'ancienneté du diabète, au taux élevés d'HbA1c et au déséquilibre tensionnel. Les facteurs de corrélation à la microangiopathie chez notre population sont l’élévation de la pression artérielle, de l'HbA1c, ainsi que la baisse du DFG ce qui rejoint les données de la littérature [9, 10].

## Conclusion

La population de patients diabétiques hypertendus est une population exposée aux complications cardiovasculaires; le présent travail retrouve une relation statistiquement significative entre l'atteinte macroangiopathique, le déséquilibre du diabète et la dyslipidémie rendant compte de la nécessité d'une prise en charge stricte de l'ensemble des facteurs de risque associés au diabète.
